# Knockout of alanine racemase gene attenuates the pathogenicity of *Aeromonas hydrophila*

**DOI:** 10.1186/s12866-019-1437-3

**Published:** 2019-04-02

**Authors:** Dong Liu, Ting Zhang, Yaping Wang, Murtala Muhammad, Wen Xue, Jiansong Ju, Baohua Zhao

**Affiliations:** College of Life Science, Hebei Normal University, Shijiazhuang, 050024 China

**Keywords:** *Aeromonas hydrophila*/ alanine racemase/ bacterial virulence

## Abstract

**Background:**

*Aeromonas hydrophila* is an opportunistic pathogen of poikilothermic and homoeothermic animals, including humans. In the present study, we described the role of Alanine racemase (*alr*-2) in the virulence of *A. hydrophila* using an *alr*-2 knockout mutant (*A.H.Δalr*).

**Results:**

In mouse and common carp models, the survival of animals challenged with *A.H.Δalr* was significantly increased compared with the wild-type (WT), and the mutant was also impaired in its ability to replicate in the organs and blood of infected mice and fish. The *A.H.Δalr* significantly increased phagocytosis by macrophages of the mice and fish. These attenuation effects of *alr*-2 could be complemented by the addition of D-alanine to the *A.H.Δalr* strain. The histopathology results indicated that the extent of tissue injury in the WT-infected animals was more severe than in the *A.H.Δalr*-infected groups. The expression of 9 virulence genes was significantly down-regulated, and 3 outer membrane genes were significantly up-regulated in *A.H.Δalr.*

**Conclusions:**

Our data suggest that *alr*-2 is essential for the virulence of *A. hydrophila.* Our findings suggested alanine racemase could be applied in the development of new antibiotics against *A. hydrophila*.

## Background

*Aeromonas hydrophila* is a water-borne Gram-negative and facultative anaerobic bacterium that is widely distributed in nature. It is an opportunistic pathogen of poikilothermic and homoeothermic animals, including humans [1]. In fish, it causes motile aeromonas septicemia (MAS), a serious disease that leads to severe economic losses [2]. In humans, *A. hydrophila* can cause acute gastroenteritis, soft tissue infections, primary septicemia, meningitis, endocarditis, pneumonia, and empyema [3]. The pathogenicity of *A. hydrophila* is complex and it produces multiple virulence factors: adhesins, cytotoxins, hemolysins, lipases, nucleases, proteases, S-layers, lateral and polar flagella, type III and typeVI secretion systems, iron-binding systems, the capacity to form biofilms and mediate virulence factor expression through quorum sensing [4–6].

Alanine racemase (Alr; EC 5.1.1.1) is a pyridoxal-5′-phosphate (PLP)-containing homodimeric enzyme that catalyzes the interconversion of L-alanine to D-alanine [7]. D-Alanine is an essential component of the peptidoglycan in the cell wall of both gram-positive and gram-negative bacteria [8]. Peptidoglycan (PG), the main component of the cell wall of bacteria, enables bacteria to endure adverse environments. Moreover, PG is also essential to maintaining cell shape and for proper growth and division [9]. The lack of alanine racemase in eukaryotes [10] makes this enzyme an attractive target for antimicrobial drug development [11, 12]. Some studies have been conducted to identify the role of alanine racemase in the pathogenicity of *Listeria monocytogenes*, *Mycobacterium tuberculosis*, *Mycobacterium smegmatis* and *Burkholderia pseudomallei* K96243 [13–16].

Recently, the molecular features of the putative alanine racemase Alr1and Alr3 (broad-spectrum amino acid racemase) from *A. hydrophila* were defined [17]. However, the biochemical features of these two racemases have not yet in-depth studied. Previously, we showed that the *E. coli alr*^−^ *dadx*^−^ double mutant MB2795 strain [18] complemented with *alr*-2 didn’t require d-alanine for growth [19]. We showed the characterization of alanine racemase (Alr-2) in *A. hydrophila* and reported the knockout of the *alr*-2 gene resulting in cell wall damage and enhanced membrane permeability under D-alanine starvation. We also developed an efficient screening method for alanine racemase Alr-2 inhibitors and identified some effective inhibitors. These inhibitors are capable of inhibiting the growth of *A. hydrophila*. These results indicated that the alanine racemase Alr-2 is important for *A. hydrophila* [20, 21]. Although Alr has been well documented as closely associated with the viability and survival of various bacteria, its physiological importance in *A. hydrophila* has not been explored. In this work, we investigate the virulence properties of the *alr*-2 deletion mutant of *A. hydrophila*. We employed both murine and fish infection models to investigate the contribution of *alr*-2 for *A. hydrophila* infection. We also check the expression level of 12 virulence-related genes to the *alr*-2 knockout mutant. The aim of this study is to explore the contribution of alr-2 to the pathogenicity of *A. hydrophila*.

## Methods

### Bacteria and culture conditions

The strains used in this study are *Aeromonas hydrophila* HBNUAh01, which was isolated from sick *Paralichthys olivaceus* [22]*,* and the alanine racemase gene (*alr*-2) knockout mutant (*A.H.Δalr*) of *A. hydrophila* HBNUAh01. *A.H.Δalr* mutant by deletion of *alr*-2 gene was constructed by homologous recombination using the suicide plasmid pK18*mobsacB* [19, 20]. Strains were cultured in Luria–Bertani (LB, Oxoid; UK) medium at 30 °C. D-alanine was obtained from Sigma (St Louis, MO, USA). Counting of *A. hydrophila* was performed by serial dilution and spread plating on LB medium. The plates were incubated at 30 °C for 48 h and colonies were counted and expressed as colony forming units (CFU)/ml. For each of the three independent experiments, two plates per dilution were used to calculate the results.

### Experimental animal

Female 4- to 6-week-old BALB/c mice (Hebei CDC, Shijiazhuang, China) were kept 5 per cage in a cabinet at 23 °C, 12 h/12 h day/night cycle; water and food were provided ad libitum throughout the experiment. The common carp, *Cyprinus carpio,* L., (100–130 g) was obtained from the fish farm of Hebei Aquaculture Experimental Station (Shijiazhuang, China). The fish were maintained at 25–26 °C in recirculating fresh water and acclimatized for 2 weeks before the experiments were performed. They were fed daily with commercial bream feed. All animal experimental procedures were strictly carried out according to the recommendations in the Guide for the Care and Use of the Laboratory Animals of Hebei Province in China. The animal experiment protocol was approved by the Animal Monitoring Committee of Hebei Normal University. All efforts were made to minimize suffering.

### Anesthesia and euthanasia

Female BALB/c mice used for the experimental procedures in this work were sacrificed by cervical dislocation after light anesthesia by intraperitoneal injection of pentobarbital (50 mg/kg). The common carp, *Cyprinus carpio,* L. were euthanized in 30-40 mg/ml buffered ethyl 3-aminobenzoate methanesulfonate (MS-222).

### Virulence assays

*A. hydrophila* WT, *A.H.Δalr* and *A.H.Δalr* supplied with 0.5 mM D-ala in LB medium (C*Δalr*) were grown at 37 °C in LB to log phase, harvested by centrifugation, washed twice, and normalized to the required inoculums in saline by adjusting the suspension to the appropriate OD_600_ value justified by the viable counts. Six groups of BALB/c mice (5 mice/ group) and 6 groups of common carp (6 carps/ group) received an intraperitoneal (i.p.) injection with 1.0 ml of suspension containing 1.5 × 10^7^ to 8 × 10^8^ CFUs of bacteria in sterile Dulbecco’s phosphate-buffered saline (DPBS). The mice and carp were observed for 14 days to determine the survival rate. LD_50_ values were calculated using SPSS 16.0 IBM modeler.

For virulence comparisons, 20 mice and 20 fish were randomly divided into 2 groups and were inoculated i.p. with 3 × 10^7^ CFUs / mouse and 3 × 10^8^ CFUs / carp of the WT or *A.H.Δalr* strain. Animals were monitored for up to two weeks to evaluate the survival rate [23].

### Macrophage-killing assays

Murine peritoneal macrophages were prepared as described [16] by peritoneal lavage of the BALB/c mice. The peritoneal exudate cells were seeded in 96-well plates at a density of 3 × 10^5^ cells per well. The macrophages were selected by adherence after 2 h of culture at 37 °C in a 5% CO_2_-humidified incubator; non-adherent cells were removed by washing with PBS and fresh RPMI 1640 medium (Sigma-Aldrich, USA) supplemented with 10% fetal bovine serum (FBS), 15 mM Hepes, 2 mM L-glutamine, 1 mM sodium pyruvate (Sigma-Aldrich), and penicillin/ streptomycin at 100 U/ml and 100 mg/ml, respectively. The wild type, *A.H.Δalr* and C*Δalr* strains were sub-cultured to OD_600_ = 0.5, collected and opsonized in RPMI 1640 medium. They were then added at a multiplicity of infection of 10 to the macrophage monolayers and incubated for 1 h to permit phagocytosis of the opsonized bacteria by the macrophages. As *A. hydrophila* were sensitive to gentamicin (Sigma-Aldrich, USA) [24], extracellular bacteria were washed two times with pre-warmed RPMI medium containing 10 μg/ml of gentamicin for 1 h. The infected macrophages were lysed with 1% Triton X-100 in PBS to allow for enumeration of the surviving bacteria on the LB agar plates.

Head-kidney (HK) macrophages from common carp were prepared as described previously [25]. The cells were distributed into 96-well tissue culture plates (1 × 10^6^ cells/well) and cultured at 22 °C in Leibovitz medium (L-15) (Thermo Scientific HyClone, China) supplemented with 10% FBS and 1% penicillin and streptomycin. Macrophages were incubated with wild type, *A.H.Δalr* and C*Δalr* strains to reach a multiplicity of infection of 10. The plates were incubated at 28 °C for 1 h. The percent survival was determined by performing serial dilutions and plating on LB media plates and calculated as (CFUs with macrophages/CFUs without macrophages) × 100%.

### Whole blood killing assays

A blood killing assay was performed as described [26] with some changes. *A. hydrophila* WT, *A.H.Δalr* and C*Δalr* were grown, washed, and resuspended in PBS. Diluted cultures of log-phase bacteria (100 μl) containing 10^3^ CFU were combined with 450 μl heparinized mouse blood, and the mixtures were incubated at 37 °C with rotation for 1 h. The percentage of live bacteria was subsequently determined by plating and calculated as follows: (CFUs after co incubation/ CFUs in original inoculums) × 100%. The negative control was made by direct inoculation of the 100 μl of PBS directly into heparinized mouse blood. Experiments were performed using blood from at least three individual mice.

### Competitive growth assays

The competitive assay was performed as described [27]. Overnight cultures of the WT and *A.H.Δalr* mutant were sub-cultured to OD_600_ = 0.5. The amount of bacteria was determined by performing serial dilutions and plating on LB media plates at 37 °C for 24 h. Both WT and *A.H.Δalr* (10^7^ CFU) were mixed together in a ratio of 1:1 and injected i.p. (0.5 ml/mice) into 6 BALB/c mice. Mice were euthanized at 24 h after inoculation, and the abdominal cavity was lavaged with 1 ml PBS. The samples were then diluted and plated onto LB agar plates. Six common carp were i.p. injected with the mixture (0.5 ml/fish). Fish were euthanized at 24 h after inoculation, and the ascites samples were diluted and plated onto LB agar plates. The percentage of *A.H.Δalr* mutants was determined by analyzing 50 colonies of each sample by PCR using the following primers designed for validating the *A.H.Δalr*: *alr*-2-up (5′-3′): CCATATGAACACAGTTACGGCCA; *alr*-2-down (5′-3′): GTTCGAACGGCCAGCTTCAACA [20]. The competitive index was determined as the *Δalr:* WT ratio in the samples.

### Determination of viable bacteria invasion in susceptible tissues

To assess the dissemination abilities of the *A. hydrophila* strains in mice and common carp, female BALB/c mice (5 mice/ group) and common carp (5 carps/ group) were challenged i.p. with the sub-cultured WT, *A.H.Δalr* and C*. Δalr* at a dose of 0.3 × 10^7^ CFUs / mouse and 1.5 × 10^8^ CFUs / carp. At 24 h following injection, the mice and common carp were euthanized, the liver, kidney, spleen, blood and ascites were collected, and the organ weights were determined. Bacterial load in the organs and samples was determined by serial dilution and plating on LB agar plates for 24 h [28].

### Histopathology

BALB/c mice were challenged i.p. with the sub-cultured WT and *A.H.Δalr* at dose of 1.7 × 10^7^ CFUs / mouse and 1.2 × 10^7^ CFUs / mouse respectively. The common carp were challenged i.p. with the sub-cultured WT and *A.H.Δalr* at a dose of 3 × 10^8^ CFUs / carp. The collected liver, kidney and spleen samples from the mice and common carp were obtained after 24 h of infection, fixed in 10% buffered formalin (PBS; pH 7.2) for 24 h, and embedded in paraffin. The animals injected with physiological saline served as negative controls. Sections (3 μm) were stained with Hematoxylin and Eosin (H&E) according to published methods [29] and scanned using an Aperio ScanScope (Aperio Technologies, Vista, CA, USA).

### Quantitative real-time PCR

To test the effect of the deletion of *alr*-2 on other significant virulence genes, quantitative real-time reverse transcription-PCR was performed to quantify the expression level of the 13 virulence genes. The virulence genes used included flagellar and pilin family protein genes (*flgE*, *flgL* and *pilB*), a major adhesion subunit gene (*cblA*), type VI secretion system-related genes (*vasH* and *traA*), a type Ш secretion system gene (*ascV*), a serine peptidase gene (*degQ*), an aerolysin gene (*aerA*), a hemolysin gene (*hlyA*), two outer membrane protein genes (*ompA* and *ompTS*) and a lipopolysaccharide (LPS) heptosyltransferase II gene (*rfaF*). The primers and method were as described [30]. Total RNA was isolated using the RNAprep pure Cell / Bacteria Kit (Tiangen, China). Total RNA concentration was determined from the absorbance at 260 nm. The first-strand cDNA was synthesized using the FastQuant RT Kit (With gDNase) kit (Tiangen, China). Controls without reverse transcription (RT) verified lack of DNA contamination. The analysis was performed on a Real-Time PCR system ABI 7500 (ABI, USA) using SYBR green I fluorescent dye. The reactions were performed in a 10 μL volume, and the cycling parameters were 95 °C for 20 s, 55 °C for 20 s, and 72 °C for 20 s at 45 cycles. Threshold cycles and dissociation curves were determined, and the gene expression levels were normalized to those of the 16S rRNA (which showed an invariant expression under the experimental conditions) [31]. ABI 7500 software v2.0.6 was used to calculate the relative expression of the mRNA target genes.

### Statistical analysis

Statistical analysis was performed with SPSS 16.0 (SPSS Inc. USA) and the Prism software program 6.0 (GraphPad Soft-ware, Inc. USA). Survival data were analyzed with the log-rank test. Except for the survival study, the *P* values in other experiments were obtained using Student’s *t* -test. *P* values < 0.05 and < 0.01 were considered statistically significant and highly statistically significant, respectively.

## Results

### *alr*-2 contributes to *A. hydrophila* pathogenesis in both mice and fish infection

*A. hydrophila* and its mutants were tested in mouse and common carp models for virulence by lethal dose 50 (LD_50_) determinations (Table 1 and Table 2). In mice, the LD50 value of the *A.H.Δalr* strain (7 × 10^7^ CFUs) was approximately 4.1-fold higher than the WT (1.7 × 10^7^ CFUs). In the common carp, the LD_50_ value of the WT strain tested was 1.4 × 10^8^ CFUs, while that of the *A.H.Δalr* was 5.1 × 10^8^ CFUs, which was 3.6-fold compared to the WT. The experiment was conducted at three separate times, and the results were the average of three experiments.

**Table 1:** Calculations of the LD_50_ values of the WT and *A.H.*Δ*alr* strains tested in mice

Dose of challenge (CFUs)	Number of deaths		Survival rate (%)	
	WT	*A.H.Δalr*	WT	*A.H.Δalr*
2.4 × 10^8^	5	5	0	0
0.9 × 10^8^	5	3	0	40
4.5 × 10^7^	5	2	0	60
3 × 10^7^	4	0	20	100
1.5 × 10^7^	2	0	60	100
LD_50_ (10^7^)	1.7	7

**Table 2:** Calculations of the LD_50_ values of the WT and *A.H.Δalr* strains tested in common carp

Dose of challenge (CFUs)	Number of deaths		Survival rate (%)	
	WT	*A.H.Δalr*	WT	*A.H.Δalr*
8 × 10^8^	6	5	0	17
5 × 10^8^	6	3	0	50
3 × 10^8^	5	1	16	83
1.5 × 10^8^	3	0	50	100
1 × 10^8^	2	0	67	100
LD_50_ (10^8^)	1.4	5.1		

**Fig. 1:**
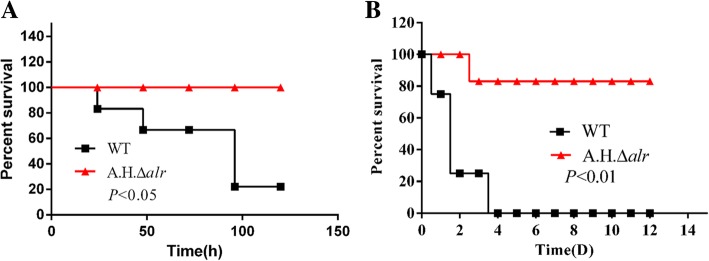
Alr-2 contributes to the virulence of *A. hydrophila*. **a** Survival curves for mice infected with WT type and *A.H.Δalr* strains by the i.p. route. **b** Survival curves for fish challenged with WT and *A.H.Δalr* strains

The virulence of the WT and *A.H.Δalr* strains was assessed with 3 × 10^7^ CFUs / mouse and 3 × 10^8^ CFUs / carp of the WT or *A.H.Δalr* strain. Most dying mice in the WT group show severe clinical signs, such as prostration depression, weakness, and anorexia. Eight out of the 10 mice died at 4 days post-infection (Fig. 1a). In contrast, all the *A.H.Δalr* infected mice survived without exhibiting any clinical signs. The mortality rate of the common carp infected with the WT strain was 100%, and death occurred at 2 days post-infection (Fig. 1b). Most dying fish showed clinical signs typical hemorrhagic septicemia, such as ulcerative lesions and skin hemorrhages. The mortality rate was 17% for the *A.H.Δalr* infected fish, and the surviving fish show no evident external lesions (Fig. 1b). These data indicate an important role of Alr-2 in *A. hydrophila* virulence.

### *alr*-2 contributes to bacterial survival in macrophages and in mouse blood

**Fig. 2:**
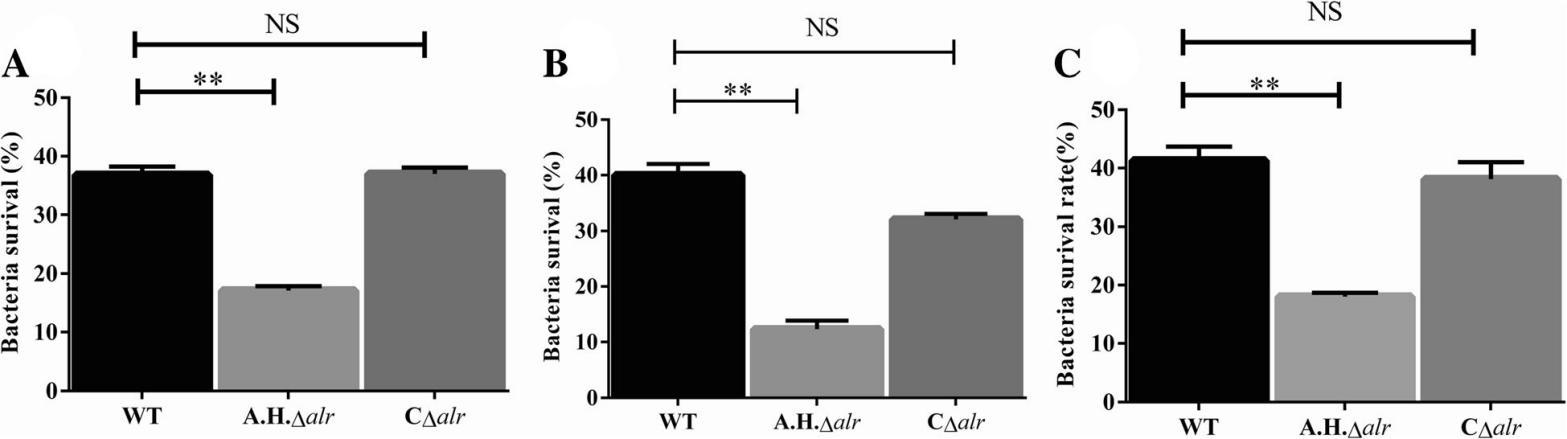
Bacterial survival in macrophages and in mouse blood. Percentage of CFUs following 1 h of incubation with mouse macrophages (**a**). Percentage of CFUs following 1 h of incubation with fish head-kidney macrophages (**b**). Percentage of CFUs following 1 h of incubation at 37 °C with heparinized mouse blood (**c**). The experiments were performed three times independently. **P < 0.01; NS: not significant

To determine if *alr*-2 promote *A. hydrophila* survival against the innate immune response, we compared the survival of the WT, *A.H.Δalr*, and C*Δalr* strains in mice and fish head-kidney macrophages. After 1 h of incubation, the *A.H.Δalr* strain was killed more efficiently than the WT strains (*P* < 0.01) (Fig. 2a and b). Next, we examined whether Alr-2 protected the WT strain in whole mouse blood. In whole blood killing assays, the survival rate of *A.H.Δalr* strain (87.7% reduction) was significantly reduced compared to WT (59.2% reduction) (*P* < 0.01) (Fig. 2c). The survival rate of complementary C*Δalr* strain is similar to that of WT (*P* > 0.05).

### *alr*-2 contributes to *A. hydrophila* survival in vivo

**Fig. 3:**
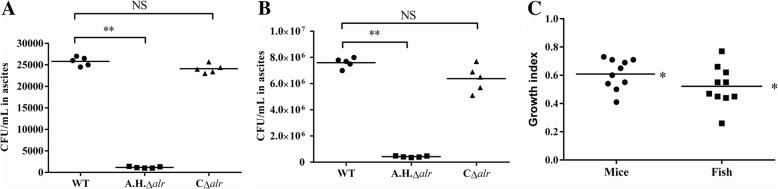
Role of Alr-2 in resistance against clearance in vivo. **a** Bacterial loads recovered from mice ascites after 24 h post-i.p. inoculation. **b** Bacterial loads recovered from fish ascites after 24 h post-i.p. inoculation. **c** In vivo competitive indices of *A.H.*Δ*alr* versus the WT strain in mouse and fish ascites. **P < 0.01; *P < 0.05; NS: not significant

In the study, ascites or hemorrhaging in the abdominal cavity were observed in mice and fish infected with *A. hydrophila*. In comparison with bacterial load in the ascites of WT-infected group, After 24 h post-inoculation, the bacterial of *A.H.Δalr* infected group was significantly lower (*P* < 0.01), while the C*Δalr* strain behaved similarly to the WT strain in the assay (*P* > 0.05) (Fig. 3a, b). The results suggest that *A.H.Δalr* could not survive effectively in vivo. We also conducted competitive growth assays in both the mouse and fish abdominal cavity model. Both the competitive indices are approximately 0.55–0.6, which is significantly less than 1 (*P < 0.05, Fig. 3c), indicating an advantage for the WT. These experiments showed that the *A.H.Δalr* strain impaired *A. hydrophila* to survival in vivo.

### *alr*-2 enhances *A. hydrophila* dissemination in multiple organs

**Fig. 4:**
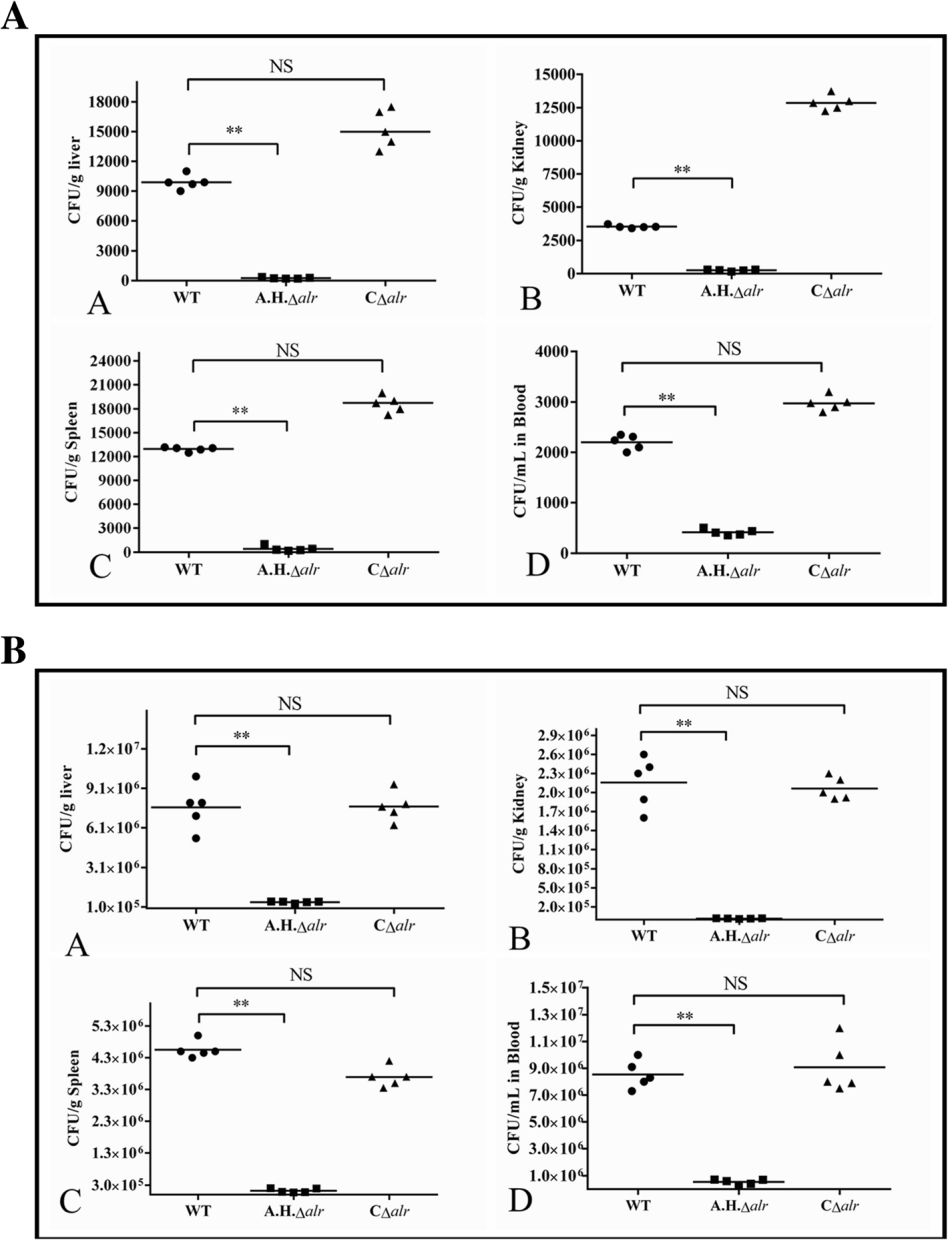
Colonization of various tissues of mice (**a**) and the common carp (**b**) by the WT, *A.H.*Δ*alr* and CΔ*alr* strains. Bacterial counts in the spleen, liver, kidney and blood were examined at 24 h post infection. The data shown are the means for the results from three independent experiments, and the bacterial cell data shown are the limits of detection. Statistical analyses were performed by a repeated measures test with a Turkey test

To assay the dissemination abilities of the WT, *A.H.Δalr* and C*Δalr* strains, three strains were inoculated i.p. into BALB/c mice and common carp, respectively. After 24 h post-infection of mice, the bacterial counts in the tissues and blood challenged with the *A.H.Δalr* were significantly lower (*P* < 0.01) than those of the WT. The concentrations of bacteria in the mice tissues and blood infected with C*Δalr* were not significantly different or higher than those of the WT (*P* > 0.05) (Fig. 4a). The changes of bacterial concentrations in the fish tissues and blood are similar to those of the mice (Fig. 4b). These results indicated that *alr*-2 promoted bacterial systemic dissemination to different organs.

### Histopathology

**Fig. 5:**
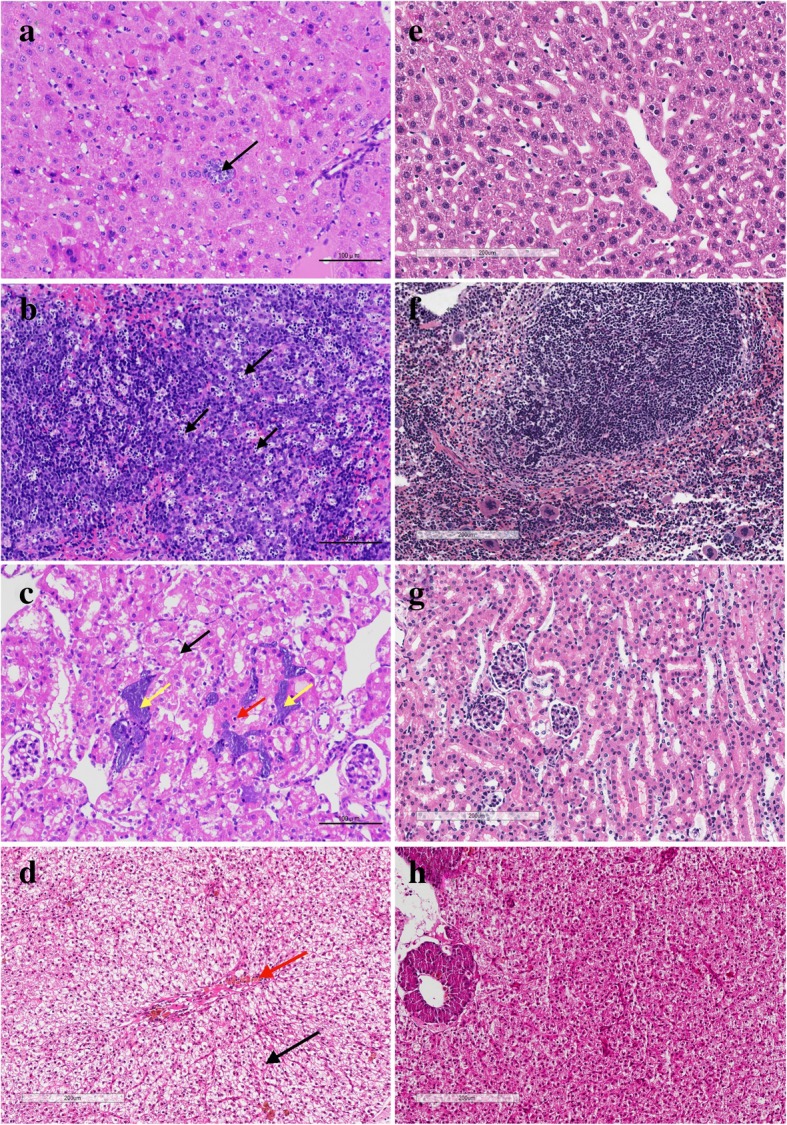
Histological examination of sampled tissues (HE-stained; Scalebar = 200 μm). **a** Liver of the mouse infected with the WT strains. **b** Spleen of the mouse infected with the WT strains. **c** Kidney of the mouse infected with the WT strains. **d** Liver of the common carp infected with the WT strains. **e** Liver of the mouse infected with *A.H.Δalr*. **f** Spleen of the mouse infected with *A.H.Δalr*. **g** Kidney of the mouse infected with *A.H.Δalr*. **h** Liver of the common carp infected with *A.H.Δalr*

Histopathological studies were carried out to examine the pathological changes in the liver, spleen and kidney of the infected mice and fish. The organ architecture of animals infected by *A.H.Δalr* (Fig. 5 panels E, F, G and H) was normal. In the group of mice and fish infected with the WT bacteria (Fig. 5, panels a, b, c and d), several pathological tissue changes were observed. The livers of the mice showed extensive and mild cell edema and deterioration, cytoplasm was loose (black arrows in Fig. 5, panel a) and occasional calcification. The spleens of the mice showed a large number of lymphocytes necrosis in the splenic nodules (black arrows in Fig. 5, panel b), irregular structures of splenic nodules and neutral expansion were observed, lymphocytosis and adjacent splenic nodules were connected. The kidneys of the mice showed renal tubular epithelial cell extensive edema and degeneration, cytoplasm loose or vacuolar (black arrows in Fig. 5, panel c), renal tubular epithelial cell necrosis, nuclear condensation (arrows in Fig. 5, panel c), cell necrosis and calcification (yellow arrows in Fig. 5, panel c). The livers of the common carp showed that the cytoplasm of the hepatocytes is vacuolated. Visible hemosiderin deposition was seen in the hepatic sinusoid and intravasculature (black and red arrows in Fig. 5, panel d).

### Expression of various virulence genes

**Fig. 6:**
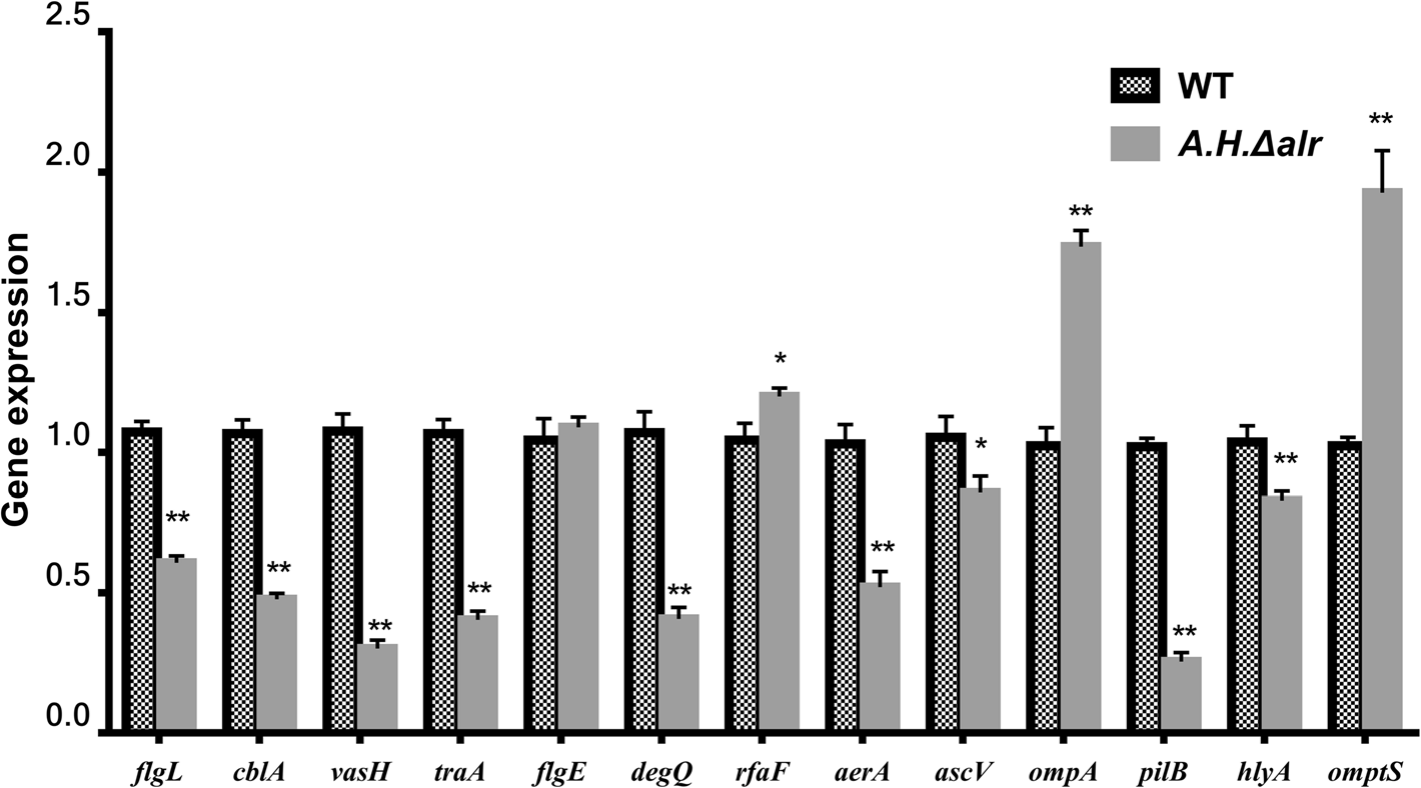
Expression studies by quantitative real-time PCR: relative transcript profiles 13 virulence-related genes of *A. hydrophila*. 16S rRNA was used as the internal control for normalization the relative expression folds of individual transcripts. Results are mean of at least three independent experiments and the standard deviation (SD) is indicated by the error bars. Significance analysis was done by Student’s t-test, *P < 0.05, **P < 0.01

Quantitative real-time PCR (RT-qPCR) showed that the expression levels of 9 genes, *flgL, pilB*, *cblA*, *vasH*, *traA*, *degQ*, *aerA*, *hlyA* and *ascV*, were significantly down-regulated in the *A.H.Δalr*. The expression of *ompA*, *ompTS* and *rfaF* was significantly up-regulated in the *A.H.Δalr*. The expression of *flgE* shows no obvious change (Fig. 6).

## Discussion

To confirm the effect of *alr*-2 on virulence, in vivo infection experiments with the mouse and common carp models were performed. Compared with the WT-infected group, the LD50 of the *A.H.Δalr* mutant was significantly increased. Survival of animals challenged with the *A.H.Δalr* was significantly increased, and the mutant also showed impaired replication in the tissues and blood. These results indicated that the *alr*-2 gene plays an important role in *A. hydrophila* virulence. These attenuation effects of *alr*-2 could be complemented by the addition of D-alanine to the *A.H.Δalr* strain.

In this study, we demonstrated that the Alr-2 deficient mutant *A.H.Δalr* lost viability in the murine peritoneal macrophages and HK macrophages growing in cell culture medium without added D-alanine compared to the WT strain. Addition of 0.5 mM D-alanine to the medium allowed *A.H.Δalr* to survive intracellularly. Some studies that have demonstrated that alanine racemase deficient mutants of *B. pseudomallei* K96243, *L. monocytogenes* and *M. tuberculosis* lost viability in murine macrophages and required exogenous D-alanine to achieve intracellular survival.

The results demonstrated that the virulence of *A.H.Δalr* was attenuated in both mice and fish, with reductions in the dissemination capacities and mortality rates. The *A.H.Δalr* mutants displayed a modest decrease in virulence which was lower than other knockout models of virulence determinants in *Aeromonas hydrophila*, summarized by Rasmussen-Ivey et al. [32]. Many proteins have proven to be important factors for the pathogenicity of *A. hydrophila*. In one study, a nuclease (*ahn*) deletion mutant was shown to attenuate the virulence of *A. hydrophila* in both fish and mice [27]. Jiang et al. reported that the histone deacetylase is an important regulatory protein contributing to the pathogenicity of *A. hydrophila* [30].

Quantitative real-time PCR showed that deletion of *alr*-2 resulted in no expression changes in *flgE*. The expression levels of the outer membrane protein (OMPS) genes (*ompA*, *ompTS*) and *rfaF* were up-regulated. In gram-negative bacteria, the *rfaF* gene is responsible for LPS biosynthesis [33]. The OMPS and LPS of the bacteria participate in maintaining bacterial cell integrity, adapting to the environment and protecting the cell from toxic compounds [34, 35]. As the knockout of the *alr*-2 of *A. hydrophila* leads to cell wall damage and enhanced membrane permeability [20], we predicted that the bacterium will produce more OMPS and LPS to stabilize the outer membrane and protect itself from toxic compounds.

The down-regulation of 9 genes, including *flgL*, *pilB*, *vasH*, *traA*, *degQ*, *cblA*, *ascV*, *hlyA* and *aerA,* is closely related to the motility, adhesion and virulence of *A. hydrophila*. *FlgL* and *pilB* are all reported to be correlated with the adhesion of *A. hydrophila* [36].

The genes of *vasH*, *traA*, *ascV, degQ*, *aerA*, and *hlyA* are all correlated with the virulence of *A. hydrophila*. The *vasH* and *ascV* mutants in *A. hydrophila* showed less toxicity and virulence in comparison with the wild-type strain [37, 38]. The serine peptidase encoded by *degQ* is used for the removal and correction of harmful proteins. This protein is an essential protein required for *E. coli* growth at high temperatures [39]. The toxins encoded by *aerA* and *hlyA*, were all major virulence factors of *A. hydrophila* [40]*.* In this study, the virulence of *A. hydrophila* to mice and fish was significantly attenuated after deletion of *alr*-2. These results are consistent with the gene expression results.

## Conclusions

We reveal that the *alr*-2 gene contributes to the virulence of *A. hydrophila* in mice and common carp. We identified that the deletion of *alr*-2 attenuates survival in macrophages of mice and fish. The *alr-*2 gene can influence the expression of 12 crucial virulence genes. Our data suggest that the *alr*-2 gene is essential for the survival and pathogenicity of *A. hydrophila*, which provides a novel target for drug design.

## Abbreviations

*A. hydrophila*: *Aeromonas hydrophila*
CFU: Colony forming units
DPBS: Dulbecco’s phosphate-buffered saline
FBS: Fetal bovine serum
H&E: Hematoxylin and Eosin
HK: Head-kidney
i.p: intraperitoneal
LD_50_: Lethal dose 50
MAS: Motile aeromonas septicemia
OMPS: Outer membrane protein
PG: Peptidoglycan
PLP: Pyridoxal-5′-phosphate
RT-qPCR: Quantitative real-time PCR
